# Children’s Cognitive Abilities Moderate Parent/Teacher Agreement on Internalizing and Externalizing Psychopathology

**DOI:** 10.1007/s10802-026-01514-2

**Published:** 2026-10-20

**Authors:** Katherine All, Caden J. Carter, Melica Kemanian Leites, Rachel Wikander, Chaojian He, Cynthia L. Huang-Pollock

**Affiliations:** Department of Psychology, The Pennsylvania State University, 140 Moore Building, 138 Fischer Road, University Park, Pennsylvania, PA 16802 USA

## Abstract

Multi-informant assessment (typically parents and teachers) is a gold-standard process used to inform diagnosis and tailor treatment in children, but multi-informant agreement is notoriously low. Children’s cognitive abilities might partially account for reporter discrepancies, particularly in situations when a child’s abilities are insufficient to meet context-specific demands (i.e., increased academic difficulties resulting in increased symptomatology at school compared to the home). Existing work examining this possibility has been limited to subjective reports of cognitive ability and/or neglected internalizing symptoms. The current study investigated whether objectively measured cognitive abilities moderate parent/teacher agreement on both externalizing and internalizing psychopathology in children. Parents and teachers of 853 school-age children (ages 8–12) rated children’s internalizing and externalizing symptoms, and children completed tasks assessing intellectual, academic, and executive functioning. Structural equation modeling was used to evaluate the latent interaction between parent-reported psychopathology and cognitive abilities when predicting teacher-reported psychopathology, thus testing whether cognitive abilities moderate parent/teacher agreement. Parent/teacher agreement on internalizing and externalizing symptoms was moderated by intellectual functioning and reading achievement, but not math skills or executive functioning. Stronger skills were associated with higher agreement, and post-hoc analyses indicated that lower cognitive abilities were associated with higher parent versus teacher concerns. Cognitive abilities moderate parent/teacher agreement, which may reflect the strength of the reporter’s knowledge of the child or reporter perceptions rather than context-based behavioral differences. Findings point to the importance of comprehensive assessment that gathers information from multiple informants and multiple domains to form a full-picture understanding of children’s functioning.

## Introduction

Multi-informant report is considered the gold standard for child clinical assessment, as it provides information on children’s behavior across multiple domains and environmental contexts. In childhood, the vast majority of waking (and sleeping) hours are spent in two primary environments: the home and the school. Consequently, parents and teachers are uniquely positioned to provide information used in diagnostic decision-making, treatment tailoring, and progress monitoring (De Los Reyes et al., 2015). However, agreement between parents and teachers is notoriously low, with meta-analyses establishing agreement ranging from 0.21 to 0.28 (Achenbach et al., 1987; De Los Reyes et al., 2015). Such discrepancies present a substantive challenge for clinicians, who must exercise clinical judgement in determining how to weigh divergent reports when evaluating symptom severity and treatment progress. While some disagreement is likely due to measurement error, differences between informants are widely acknowledged to represent clinically meaningful information (De Los Reyes, 2011; De Los Reyes & Kazdin, 2005). Research to better understand the causes of such discrepancies is therefore crucial to provide clinicians with empirically grounded guidance for interpreting and reconciling multi-informant reports.

Parental psychopathology and demographic characteristics of the child and family are among the most widely studied contributors to informant discrepancies. The presence of parental depression or stress (Briggs-Gowan et al., 1996; Harvey et al., 2013; van der Oord et al., 2006; Yeguez & Sibley, 2016; Youngstrom et al., 2000), children belonging to racially/ethnically minoritized backgrounds (De Los Reyes & Kazdin, 2005; Harvey et al., 2013; Lau et al., 2004; Youngstrom et al., 2000), lower socioeconomic status (Lawson et al., 2017; Stone et al., 2013), and larger household size (Castagna et al., 2020; Harvey et al., 2013) are all known to exacerbate parent/teacher reporting differences. A more limited body of work has investigated teacher characteristics such as gender, age, and teaching experience as moderators (Berg-Nielsen et al., 2012; Carneiro et al., 2019; Tian et al., 2025), although effect sizes are variable.

Such research posits that divergent ratings of child behaviors may reflect differences in rater perceptions or attributions of behavior based on the child or rater’s demographic characteristics. For example, some have argued that racial/ethnic differences in multi-informant agreement reflect rater bias or cross-cultural differences in behavioral norms (Lau et al., 2004). Others have proposed that informants make different attributions about the cause of a child’s behavior and necessity of treatment, which in turn impacts the memories they access while completing questionnaires on the child (De Los Reyes & Kazdin, 2005). For instance, a teacher may believe that a child’s withdrawn behavior is caused by struggles with schoolwork and therefore does not warrant treatment, leading to lower endorsements on internalizing symptoms.

However, another prominent and non-mutually exclusive view is that parent/teacher discrepancies accurately reflect different behaviors across home/school contexts (De Los Reyes, 2011). Each context presents unique demands, social influences, and consequences that would be expected to shape behavioral expression (Harvey et al., 2013; Heiervang, 2001). For example, a child with greater cognitive struggles might demonstrate more externalizing or internalizing behaviors in the context of school, when demands are highest, compared to the home. If this is the case, then individual differences in fundamental cognitive abilities would be expected to predict a child’s capacity to meet these context-specific demands. Capturing such differences could thus help reconcile low interrater agreement.

Of the primary cognitive capacities, academic, intellectual, and executive functioning are strong candidate abilities to consider. They are known to be transdiagnostic risk factors for psychopathology (Huang-Pollock et al., 2017; Kavish et al., 2020; Monzonís-Carda et al., 2024; Zhang et al., 2019) and predict worse trajectories in psychological and broader life functioning (Stucke & Doebel, 2024). Relatively little research has tested these cognitive abilities as moderators of parent/teacher agreement. Some have examined parent/teacher *ratings* of cognitive ability (Deng et al., 2004; González-Lorza et al., 2025; Mctiernan, 2019), but such designs introduce significant confounds due to the presence of shared method variance. That is, it is unclear whether findings reflect the true impact of cognitive ability on parent/teacher agreement or merely raters’ *perceptions* of cognitive ability, which may be linked to their overall perception of the child and, thus, their ratings of behavior problems.

To our knowledge, only four studies have investigated the moderating role of individual cognitive abilities using objective measures of performance (See Table 1). Three found no moderating effect of reading/math achievement, intellectual functioning, or inhibitory control on parent/teacher agreement for externalizing behaviors (Chi & Hinshaw, 2002; Lavigne et al., 2015; Takeda et al., 2020). However, Rommelse and colleagues (2017) found that intellectual functioning was associated with *stronger* agreement on broad-band internalizing symptoms in all children and broad-band externalizing symptoms in boys (but not girls).

**Table 1:** Studies assessing the role of cognitive abilities in parent/teacher agreement on psychopathology

Study	Sample type	Cognitive ability	Symptoms assessed	Effect
Takeda et al. (2020)	Referred children ages 4–18 at an ADHD evaluation center *N* = 1,364	Academic Achievement (reading and math skills assessed via the Wechsler Individual Achievement Test; parent report of homework performance, history of special education, grade failure) Intellectual Functioning (full-scale IQ; Wechsler Intelligence Tests – multiple versions)	Externalizing Symptoms (ADHD-RS-IV, home and school versions)	Stronger homework performance, but no other measures of academic performance or intellectual functioning, was associated with *higher* parent/teacher agreement on symptoms of ADHD
Chi and Hinshaw (2002)	6-10-year-old children with ADHD diagnoses *N* = 96	Intellectual Functioning (verbal IQ; Wechsler Intelligence Scale for Children, third edition)	Externalizing Symptoms (Conners Rating Scales) Combined Symptoms (Total Behavior Problem scores on CBCL/TRF)	No effect found
Rommelse et al.(2017)	Community sample of Dutch children ages 10–12 years old *N* = 2,221	Intellectual Functioning (full-scale IQ; Revised Wechsler Intelligence Scales short-form)	Externalizing Symptoms (broadband externalizing, attention problems and hyperactivity/impulsivity scales from the CBCL/TRF) Internalizing Symptoms (broadband internalizing scale from the CBCL/TRF)	Higher intellectual functioning was associated with *higher* agreement on internalizing symptoms in all children and externalizing symptoms in boys (but not girls).
Lavigne et al. (2015)	Community sample of 4-year-old children *N* = 344	Executive Functioning (statue subtest from the Developmental Neuropsychological Assessment)	Externalizing Symptoms (ODD scale of Child Symptom Inventory)	No effect found

Sparse research and limitations in study design make it difficult to construct a strong take-away message. Methodologically, all four studies used multiple regression analyses to predict discrepancy scores on single questionnaires (e.g., the Child Behavior Checklist or Behavior Assessment System for Children) and conducted separate analyses for each scale (e.g., separate regressions for broadband externalizing, attention problems and hyperactivity/impulsivity scales). The use of dimensional discrepancy scores as outcomes has been criticized in the literature (Laird & De Los Reyes, 2013; Laird & Weems, 2011), and analyzing one scale at a time risks measuring the interrater reliability (i.e., the alignment of the parent/teacher versions of each specific scale) rather than parent/teacher agreement on overall psychopathology symptoms. A better approach would be to represent psychopathology symptoms as latent variables using structural equation modeling (SEM), which allows for the integration of multiple subscales across multiple measures, thus assessing agreement on overall symptomatology rather than agreement on specific scales. The use of latent variables would also allow models to partition out measurement error, resulting in more reliable measures of psychopathology (Bollen & Noble, 2011).

Furthermore, all but one of the aforementioned studies focused on externalizing behaviors such as hyperactivity, aggression, or attention problems. This emphasis likely reflects the more disruptive nature of externalizing symptoms, which often elicits greater concern, and the necessity of multi-informant report for diagnoses such as attention deficit/hyperactivity disorder (ADHD). Ironically, parent/teacher agreement is known to be lower for internalizing compared to externalizing behaviors (De Los Reyes et al., 2015), and some internalizing experiences (e.g., social anxiety) are by definition more prominent in non-home environments (e.g., giving class presentations, interacting with peers). Therefore, despite the historic focus of study into the moderators of parent/teacher agreement for externalizing behaviors, identifying moderators of agreement for internalizing concerns is arguably just as pressing.

The current study aims to investigate whether cognitive abilities moderate parent/teacher agreement on externalizing and internalizing symptoms in children. While academic, intellectual, and executive functioning are all correlated constructs, they are not unitary (Ackerman et al., 2005; Cortés Pascual et al., 2019) and may differentially impact parent/teacher agreement. To avoid conflating cognitive ability with informant perceptions, we utilized performance-based measures of ability. Because the school environment places a higher demand on academic skill compared to the home, we hypothesized that academic achievement would moderate parent/teacher agreement. Specifically, children with lower academic achievement may struggle more at school and therefore receive higher ratings of behavioral concern from teachers compared to parents, leading to weaker cognitive abilities being associated with lower parent/teacher agreement.

Potential mechanisms for intellectual and executive functioning are less clearcut. Unlike academic achievement, which is presumably more central to success at school, both intellectual and executive functioning are higher order cognitive processes critical for successful adaptive functioning across contexts, so it is not clear that either would drive true differences in behavior. However, it is possible that teachers may be more aware of a child’s general cognitive abilities than parents, given more formal and frequent measures of ability (e.g., assignments or tests) and having a larger population against which to judge (i.e., classmates). Therefore, if intellectual or executive functioning were to moderate parent/teacher agreement, it would likely be via altered informant perceptions, in a similar direction as achievement.

## Methods

### Participants

Data was collected from 853 children (54.40% males) ages 8–12 (*M* = 9.60) in the northeastern United States from 2005 to 2015. Reflecting regional demographics, the sample race and ethnicity breakdown, as reported by parents, was 76.31% White/non-Hispanic, 5.86% Black/non-Hispanic, 4.34% White/Hispanic, 0.70% Black/Hispanic, 0.94% other Hispanic, 1.52% Asian, 5.86% Mixed, and 4.47% other or unknown. The reported income breakdown was 10.55% <$30,000, 25.79% $30–60,000, 25.56% $60–90,000, 31.89% >$90,000, and 6.21% unknown. According to parent report, 2.70% of children were currently prescribed short-acting stimulants, 14.07% long-acting stimulants, 0.12% non-stimulant ADHD medication (e.g., Tennex, Straterra), 81.13% no ADHD medication, and 1.99% unknown. Additionally, 1.06% were prescribed psychological medication for a mood disorder. All children were in regular education classrooms; 22.63% received services for learning problems according to parent report.

### Procedure

Participants were recruited as part of a larger study investigating the cognitive mechanisms of attention problems in children. Community recruitment via posted flyers, internet posts, and advertisements through newspaper and radio targeted families from the Centre, York, and Dauphin counties of Pennsylvania, and participating families were asked for their child’s teacher’s contact information. Children presenting with high inattention, hyperactivity, and impulsivity also commonly present with co-occurring internalizing and externalizing disorders (Reale et al., 2017), ensuring a wide range of both internalizing and externalizing symptomology.

Both typically developing children and children whose parents and teachers reported elevated problems with attention and/or hyperactivity were screened into the study. Children were excluded from participation if they (a) were currently prescribed and taking a non-stimulant psychoactive medication that could not be washed out prior to participation, (b) had a parent-reported autism spectrum disorder, intellectual or sensorimotor disability, psychosis, or neurological disorder, or (c) were in a temporary custody situation with an uncertain outcome.

Parents and teachers completed questionnaires about their children’s psychological functioning, followed by an in-person assessment session with families. During this visit, parents completed structured clinical interviews administered by a trained research assistant, while children completed a battery of questionnaires and performance-based tasks designed to assess intellectual, academic, and executive functioning. All procedures were completed in compliance with human subjects approval from Pennsylvania State University Institutional Review Board (IRB#32126), and informed written consent from parents and verbal assent from children were obtained prior to participation.

### Measures

#### Psychopathology Symptoms

See Table 2 for description of sample symptomatology.

**Table 2:** Psychopathology measure distributions

	Parent Report			Teacher Report		
	Mean (SD)	Min	Max	Mean (SD)	Min	Max
BASC						
Hyperactivity	55.85 (14.77)	34.00	103.00	53.22 (12.41)	41.00	91.00
Aggression	51.59 (11.18)	37.00	95.00	49.83 (9.99)	43.00	100.00
Conduct Problems	52.63 (12.18)	37.00	108.00	49.28 (9.31)	42.00	95.00
Anxiety	52.92 (12.82)	28.00	100.00	49.59 (11.10)	38.00	103.00
Depression	54.04 (12.85)	35.00	118.00	51.34 (11.37)	41.00	106.00
Withdrawal	51.66 (11.58)	35.00	100.00	51.07 (10.89)	38.00	97.00
Conners						
Hyperactivity	59.13 (15.42)	40.00	90.00	54.47 (12.50)	40.00	90.00
Aggression	54.61 (13.07)	39.00	90.00	52.75 (11.67)	45.00	90.00

BASC and Conners are standard scores with *M =* 50

##### Behavior Assessment System for Children

Parents and teachers completed the Behavior Assessment System for Children (BASC; Reynolds, 2010), a broad band questionnaire that assesses internalizing, externalizing, and adaptive behavior in children. The BASC is a well-normed instrument that has demonstrated strong internal consistency (coefficient alphas ranging from 0.80 to 0.86 for individual scales; Reynolds, 2010). Informants rated items as being true of the child “never,” “sometimes,” “often,” or “always.” The BASC has a version normed for children (ages 6–11) and a version normed for adolescents (ages 12–18). Furthermore, the BASC-2 was updated by Pearson Clinical in 2015, meaning some informants completed the BASC-2 and others completed the BASC-3 depending on year of participation. All four versions of the BASC (2/3, child/adolescent) were used to maximize sample size. Subscale standard scores from the Hyperactivity, Aggression, and Conduct Problems scales were used for externalizing symptoms, and standard scores from the Anxiety, Depression, and Withdrawal scales were used for internalizing symptoms.

##### Conners Rating Scale

Parents and teachers also completed the Conners Rating Scale, a questionnaire commonly used in the assessment of ADHD and other externalizing disorders that has demonstrated strong psychometric properties, including strong internal consistency ($\:\alpha\:$ ranging from 0.77 to 0.97; mean$\:\alpha\:=.90$), test-retest reliability (* r* ranging from 0.71 to 0.98; mean * r=.83*), and inter-rater agreement between informants (* r* ranging from 0.52 to 0.94; mean * r=.77*; Conners, 2003). Informants rated statements about children on a 4-point scale, ranging from “Not at all true” to “Very much true.” Parents completed the long form, and teachers completed the short form; participants completed the Revised or 3rd edition of the scale depending on the year they were recruited. Subscale standard scores from the Hyperactivity and Aggression scales were used for externalizing symptoms.

##### **Diagnostic Interview Schedule for Children**

A trained research assistant administered all modules of the Diagnostic Interview Schedule for Children (DISC; Shaffer et al., 2000) to parents under the supervision of a licensed clinical psychologist. The DISC is a structured clinical interview developed by the National Institute of Mental Health for clinical research purposes that assesses for over 30 psychiatric disorders including anxiety, mood, and disruptive behavior disorders. The DISC exhibits test-retest reliability ranging from 0.43 to 0.96 (Shaffer et al., 2000). The sample’s diagnostic demographic information was calculated based on a past-year diagnosis of any given disorder as determined by DSM-5 criteria. See Table 3 for diagnostic information.

**Table 3:** Diagnostic information

Diagnosis	*N*	Percent of Sample
ADHD	424	49.71%
ODD/CD	200 / 42	23.45% / 4.92%
GAD	60	7.03%
MDD/PDD	18 / 6	2.11% / 0.70%
Co-Occurring Diagnoses	188	22.04%

Diagnoses calculated based on a past-year diagnosis of any given disorder as determined by DSM-5 criteria measured via the DISC. Total *N* = 853. *ADHD =* attention/deficit-hyperactivity disorder, *ODD =* oppositional defiant disorder, *CD =* conduct disorder, *GAD =* generalized anxiety disorder, *MDD =* major depressive disorder, *PDD =* persistent depressive disorder, Co-Occurring Diagnoses = children who met criteria for more than one past-year diagnosis

#### Intellectual Functioning

Children’s IQ was estimated using a two-subtest short-form of the Wechsler Intelligence Scale for Children (WISC-IV; test-retest reliability = 0.93, predictive validity = 0.87; Sattler & Dumont, 2004; Wechsler, 2003). Trained research assistants administered the Vocabulary and Matrix Reasoning subtests, in which children were asked to define verbally presented words and complete visual patterns and matrices respectively. Both subtests display strong internal consistency (α = 0.89 for both) and high test-retest reliability (*r* = .92 for Vocabulary; *r* = .85 for Matrix Reasoning; Wechsler, 2003). A short-form composite that did not include working memory subtests was specifically selected so as not to contaminate measures of IQ and executive functioning. See Table 4 for means and correlations of all measures of cognitive abilities.

**Table 4:** Cognitive ability measure distributions and correlations

	M (SD)	Range	SFIQ	WR	NO	DSB	LNS
Estimated IQ	105.28 (14.39)	57–152					
Word Reading	103.98 (14.48)	46–136	0.59				
Numerical Operations	103.79 (19.58)	42–160	0.33	0.29			
Digit Span Backward	10.07 (2.77)	1–19	0.37	0.41	0.23		
Letter Number Sequencing	10.66 (2.58)	1–18	0.54	0.52	0.30	0.40	
Finger Windows Backward	9.33 (3.33)	1–18	0.24	0.18	0.15	0.20	0.31

Intellectual and academic functioning measures are standard scores with *M* = 100. Executive functioning measures have *M =* 10. All correlations significant at *p* < .001. SFIQ = estimated IQ (two-subtest short-form of WISC-IV), WR = WIAT-III word reading standard score, *NO = WIAT-III* numerical operations standard score, *DSB = * WISC-IV digit span backward score, LNS = WISC-IV letter number sequencing score, Finger Windows Backward = WRAML-2 finger windows backwards adaptation

#### Academic Functioning

Children’s academic achievement was assessed using subtests of the Wechsler Individual Achievement Test 3rd Edition (WIAT-III; Wechsler, 2009) administered by a trained research assistant. Children read words from a list (Word Reading; internal consistency $\:\alpha\:=.97$, test-retest * r=.93*) and solved written math problems involving basic arithmetic (Numerical Operations; internal consistency $\:\alpha\:=.93$, test-retest * r=.87*). Standard scores were obtained for Word Reading and Numerical Operations. Academic achievement in reading and math were treated as separate in analyses, rather than forming an overall academic achievement factor, due to the constructs’ weak association in the current sample (*r* = .29) and theoretical recommendations against two-indicator factors in the absence of a relatively narrow construct.

#### Executive Functioning

Executive functioning was operationalized as working memory, a prototypical executive function that reflects general executive ability (Huang-Pollock et al., 2017). Working memory was assessed using tasks adapted from the Working Memory Index of the WISC (overall WMI composite $\:\alpha\:=.92$, test-retest $\:r=.89;\:$Wechsler, 2014) and the WRAML-2 (subtest internal consistencies $\:\alpha\:=.86$ to * .93*; Sheslow & Adams, 2003). From the WISC, children completed the letter-number sequencing task (internal consistency $\:\alpha\:=.90$, test-retest * r=.83*), in which they listened to mixed numbers and letters and repeated them back in ascending/alphabetical order, and the digits backwards task (internal consistency $\:\alpha\:=.80$, test-retest * r=.74*), in which they listened to a trained research assistant read a series of digits at a rate of one digit per second and were asked to recall the digits out loud in reverse order. Finger Windows Backwards was adapted from the Finger Windows Forwards subtest of the WRAML-2 (Sheslow & Adams, 2003) to assess working memory as opposed to short-term memory (Nigg, 2006) and has been used extensively as a measure of working memory in children (Hlutkowsky et al., 2026; Lambek et al., 2011; Manassis et al., 2007; McQuade et al., 2013). A trained research assistant placed the tip of a pencil through holes (“windows”) in an opaque plastic board at the rate of one per second, and children were asked to place their finger in the holes in reverse order. Backwards versions of the digit span and finger windows tasks were used to specifically assess working memory. That is, children were required to remember *and manipulate* information, rather than simply recall information verbatim.

### Data Analytic Strategy

Structural equation modeling (SEM) using the R lavaan (Rosseel, 2012) and semTools (Jorgensen et al., 2025) packages was used to evaluate the latent interaction between parent-reported psychopathology and cognitive abilities when predicting teacher-reported psychopathology. Latent variable modeling allows for the separation of measurement error from latent constructs, resulting in more accurate estimates of relationships between variables (Bollen & Noble, 2011), and has been recommended as a better representation of the dimensional nature of psychopathology (Kotov et al., 2017). An interaction approach was used to model the effect of cognitive abilities on parent/teacher agreement because it incorporates the severity of the reported behavior and avoids common pitfalls in interpretation of difference-score models (see Laird and Weems (2011) for review).

Model fit was assessed by inspecting the 𝜒2 test (although see Bentler and Bonett (1980) for oversensitivity to large sample sizes), the Comparative Fit Index (CFI), the Tucker-Lewis Index (TLI), Root Mean Square Error of Approximation (RMSEA), and the Standardized Root Mean Square Residual (SRMR). Robust CFI, TLI, and RMSEA were used to obtain more accurate fit indices in the presence of non-normality, which is useful for skewed distributions such as psychopathology symptoms (Brosseau-Liard & Savalei, 2014). We considered CFI ≥ 0.95, TLI ≥ 0.95, RMSEA ≤ 0.05, and SRMR ≤ 0.08 to indicate good fit, and CFI ≥ 0.90, TLI ≥ 0.90, RMSEA ≤ 0.10, and SRMS ≤ 0.10 to indicate acceptable fit (Bentler & Bonett, 1980; Chen et al., 2008; Hu & Bentler, 1999).

Missing data was less than 2.00% for all variables with the exception of the finger windows backwards task, which was missing for 27.43% of subjects. Missing data was not related to any variables or constructs of interests besides parent-reported hyperactivity on the Conners; children who were missing finger windows backwards displayed slightly lower (*M* = 56.39, *SD* = 14.48) parent-reported hyperactivity compared to children who were not missing the task (*M* = 60.15, *SD* = 15.64). The pattern of findings did not change when finger windows backwards was removed from the executive functioning variable.

First, measurement models were created with factors for parent-reported psychopathology, teacher-reported psychopathology, and cognitive abilities. Separate models were run for internalizing and externalizing symptoms, as well as the four potential moderators (reading and math skills split), resulting in a total of eight models. In the externalizing models, covariances were introduced to account for the additional variance shared between the BASC hyperactivity and Conners hyperactivity subscales, as well as the BASC aggression and Conners aggression subscales, as indicated by modification indices. All latent variables were allowed to correlate.

Next, structural models with parent-reported psychopathology and cognitive abilities predicting teacher-reported psychopathology were conducted. These models evaluated the main effects of parent-report and cognitive abilities before an interaction was introduced. All models used a robust maximum likelihood estimator, which is robust to normality violations in the data. Model fit was evaluated at this stage, prior to the interaction, as is recommended by Schoemann and Jorgensen (2021).

Finally, latent interaction variables were created using an all pairs product-indicator approach. In such approaches, each indicator of the latent predictor is multiplied by each indicator of the latent moderator, resulting in a set of products that are then used as indicators of a new latent variable representing the interaction. Double mean-centering, in which predictor and moderator indicators are mean-centered prior to calculating product indicators, and product indicators are then additionally mean-centered, was used as recommended in the literature (Lin et al., 2010; Schoemann & Jorgensen, 2021). Residual covariances were estimated between product indicators that shared a first-order indicator, with equality constraints applied as discussed in Schoemann and Jorgensen (2021). For models that included variables with vastly different scales, predictor and moderating variables were scaled using z-score methods prior to double mean-centering.

If the latent interaction factor was significant, the interaction was further probed and plotted at one standard deviation (SD) below the moderator, the mean of the moderator, and one SD above the moderator. All models were initially estimated using standardized latent variables for ease of interpretation of factor loadings. The models were re-estimated allowing the latent variances to be freely estimated to compute the factor standard deviations for simple slopes analysis.

## Results

Prior to estimating regression and interaction effects, parent/teacher report of internalizing and externalizing symptoms were combined in a single model to examine reporter agreement. As expected, agreement for externalizing behaviors (*r* = .59) was significantly higher than agreement for internalizing behaviors (*r* = .39), Δχ²(1) = 31.52, *p* < .001. See Table 5 for all correlations.

**Table 5:** Latent factor correlations

	Parent internalizing	Parent externalizing	Teacher internalizing
Parent Internalizing			
Parent Externalizing	.67		
Teacher Internalizing	.39	.41	
Teacher Externalizing	.29	.59	.58

All correlations significant at p < .001

The main effects models for both externalizing and internalizing symptoms fit reasonably well (See Table 6 for fit statistics, Table 7 for model coefficients). When considering CFI and SRMR, which do not penalize for model complexity, all models met the SRMR cut-off of < 0.08 for adequate model fit. However, the internalizing models for intellectual functioning, reading skills, and math skills had CFI values that fell 0.01 short of the conventional cut-off of > 0.90. This weaker fit may reflect the lower correlations among the internalizing subscales, which may have resulted in less well-defined internalizing factors. However, when analyses were repeated using manifest variables (i.e., individual depression, anxiety, and withdrawal subscales), the interpretation of results did not change.

**Table 6:** Goodness of fit indices

Model	Indices				
	𝜒2	RMSEA	CFI	TLI	SRMR
Internalizing					
Intellectual Functioning	𝜒2(12) = 187.85	0.145	0.89	0.80	0.058
Reading Skills	𝜒2(12) = 181.94	0.145	0.89	0.80	0.058
Math Skills	𝜒2(12) = 180.37	0.143	0.89	0.81	0.057
Executive Functioning	𝜒2(24) = 169.07	0.104	0.90	0.85	0.050
Externalizing					
Intellectual Functioning	𝜒2(38) = 380.01	0.118	0.94	0.92	0.064
Reading Skills	𝜒2(38) = 356.48	0.115	0.95	0.92	0.063
Math Skills	𝜒2(38) = 366.95	0.116	0.95	0.92	0.064
Executive Functioning	𝜒2(58) = 363.23	0.103	0.94	0.92	0.068

*RMSEA*, *CFI*, and *TLI* are robust indicators. *RMSEA =* Root Mean Square Error of Approximation, *CFI =* Comparative Fit Index, *TLI* = Tucker-Lewis Index, *SRMR =* Standardized Root Mean Square Residual

**Table 7:** Model regression coefficients

	Main Effects		Interaction
	Parent β (SE)	Moderator β (SE)	β (SE)
Internalizing			
Intellectual Functioning	**0.42 (0.05)**, ***p*** **< .001**	-0.03 (0.04), *p* = .492	**0.16 (0.06)**, ***p*** **= .002**
Reading Skills	**0.41 (0.05)**, ***p*** **< .001**	**-0.10 (0.04)**, ***p*** **= .008**	**0.10 (0.05)**, ***p*** **= .035**
Math Skills	**0.42 (0.05)**, ***p*** **< .001**	**-0.06 (0.03)**, ***p*** **= .040**	0.02 (0.04), *p* = .660
Executive Functioning	**0.40 (0.06)**, ***p*** **< .001**	**-0.16 (0.06)**, ***p*** **= .002**	0.11 (0.07), *p* = .076
Externalizing			
Intellectual Functioning	**0.59 (0.06)**, ***p*** **< .001**	-0.01(0.04), *p* = .722	**0.11 (0.05)**, ***p*** **= .007**
Reading Skills	**0.59 (0.06)**, ***p*** **< .001**	-0.03 (0.04), *p* = .372	**0.08 (0.05)**, ***p*** **= .052**
Math Skills	**0.60 (0.06)**, ***p*** **< .001**	-0.03 (0.04), *p* = .318	0.01 (0.06), *p* = .792
Executive Functioning	**0.54 (0.07)**, ***p*** **< .001**	**-0.14 (0.06)**, ***p*** **= .004**	0.06 (0.06), *p* = .161

Main effects and interaction coefficients are derived from separate models predicting teacher-report of psychopathology. β represents the standardized beta coefficient

**Fig. 1:**
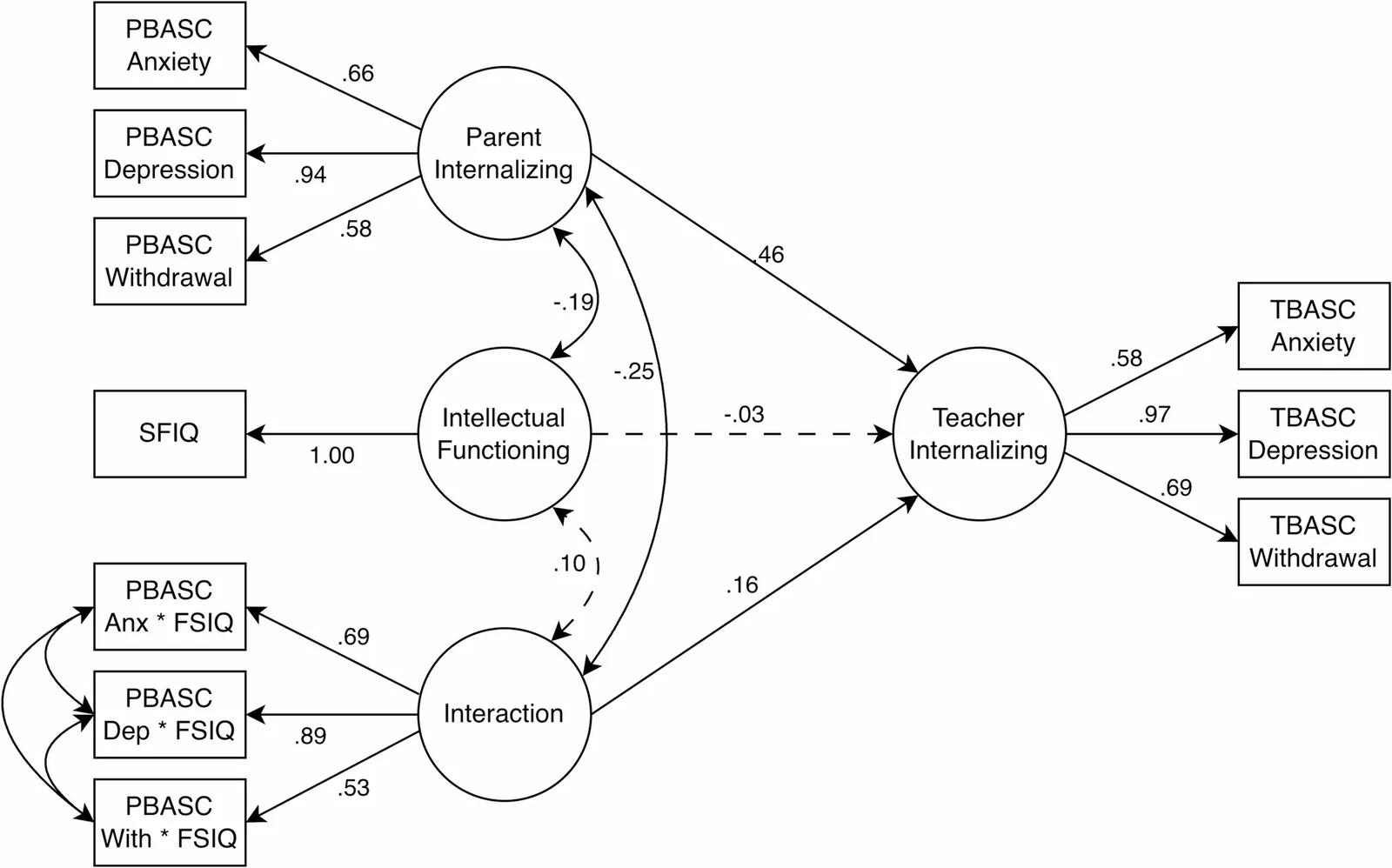
Latent interaction model between parent-reported internalizing symptoms and intellectual functioning when predicting teacher-reported internalizing symptoms. Solid lines represent significant relationships, and dotted lines represent nonsignificant relationships. PBASC = Subscale standard scores on Behavior Assessment System for Children (parent-report). TBASC = Subscale standard scores on Behavior Assessment System for Children (teacher-report). SFIQ = Two-subtest short-form composite score on the Wechsler Intelligence Scale for Children, 4th edition

For internalizing behaviors, estimated intellectual functioning significantly moderated the relationship between parent-report and teacher-report of internalizing symptoms, β = 0.16, *SE* = 0.06, *p* = .003 (Fig. 1). Probing the interaction revealed that parent-report was significantly associated with teacher-report at all levels of estimated intellectual functioning, but this relationship increased as estimated intellectual functioning increased. That is, parents and teachers demonstrated higher agreement on children’s internalizing behaviors when children’s estimated intellectual functioning was stronger. Academic achievement in reading also moderated parent/teacher agreement in a similar pattern, β = 0.10, *SE* = 0.05, *p* = .035. Academic achievement in math and executive functioning did not moderate agreement.

For externalizing behaviors, estimated intellectual functioning, β = 0.11, *SE* = 0.05, *p* = .007, moderated parent/teacher agreement in the same direction. Academic achievement in reading followed the same pattern, β = 0.08, *SE* = 0.05, *p* = .052, but became nonsignificant when additional covariates were introduced. Academic achievement in math and executive functioning were not significant moderators.

When gender, medication status (yes/no; parent-report), learning services (yes/no; parent-report), and psychiatric diagnosis (yes/no; DISC) were considered as potential covariates, the pattern of results reported above did not change, with the exception of academic achievement in reading and externalizing symptoms, which became non-significant when diagnosis and the receipt of learning services were added.

### Post-Hoc Analyses

We next attempted to answer whether, in the context of lower cognitive ability, parent/teacher disagreement was being driven by higher report from parents or teachers. To answer this question, we extracted factor scores for total parent and teacher externalizing and internalizing behaviors. We then divided participants into three groups, those with higher parent report, higher teacher report, and roughly equivalent report, and compared the groups on the moderator variable of interest^1^. We hypothesized that lower cognitive abilities would be specifically associated with the *higher teacher* report group, given the assumption that lower cognitive abilities would lead to more struggles in the school compared to home environment.

Post-hoc analyses were conducted only for the models in which there was a significant interaction (Table 8). For internalizing symptoms, estimated intellectual functioning significantly differed among the three groups, *F*(2, 848) = 3.33, *p* = .036. Post-hoc Bonferroni pairwise comparisons indicated that children for whom parents reported more internalizing behaviors than teachers (higher parent) had lower estimated intellectual functioning than children with roughly equal parent/teacher report, *p* = .032. The three groups did not significantly differ on reading achievement, *F*(2, 845) = 2.24, *p* = .12.

**Table 8:** Post-hoc mean ability comparisons

	Internalizing Problems	Externalizing Problems	
	Intellectual Functioning Mean (SE)	Intellectual Functioning Mean (SE)	Word Reading Mean (SE)
Higher Parent	102.70 (1.12)*	101.46 (1.05)*	100.18 (1.05)*
Roughly Equal	105.97 (0.62)*	106.96 (0.64)*	105.69 (0.65)*
Higher Teacher	105.66 (1.16)	104.55 (1.09)	103.15 (1.10)

Factor score differences ranged from − 4.62 to 3.79 for internalizing problems and − 3.98 to 3.12 for externalizing problems. Scores ranging from − 0.70 to 0.70 for internalizing, and − 0.50 to 0.50 for externalizing, were considered roughly equivalent in order to ensure sufficient participants in each group. Findings held when the range was lowered. Only abilities that differed significantly in initial ANOVA comparisons are presented. * Indicates means within a column that were significantly different at *p* < .05 in Bonferroni-corrected post-hoc comparisons

Externalizing symptoms followed a similar pattern; estimated intellectual functioning differed among the groups, *F*(2, 848) = 10.28, *p* < .001, and children with higher parent report had lower estimated intellectual functioning than children with roughly equal parent/teacher report, *p* < .001. For externalizing symptoms, reading skills did differ significantly among the groups, *F*(2, 845) = 10.31, *p* < .001, once again such that children with higher parent report demonstrated lower reading skills than those with roughly equivalent report, *p* < .001.

Thus, contrary to expectations, discrepancies in parent/teacher agreement in the context of lower cognitive ability were driven by higher *parent* report of symptoms.

## Discussion

The present study investigated the impact of cognitive ability on parent/teacher agreement on externalizing and internalizing symptomatology in children. Using performance-based measures of academic, intellectual, and executive functioning, we found that individual differences in estimated intellectual functioning and reading achievement moderated agreement for internalizing and externalizing concerns. Academic achievement in mathematics and executive functioning were not significant moderators. In all cases, our hypotheses regarding directionality were confirmed; higher cognitive abilities were associated with higher parent/teacher agreement.

Because intellectual ability and executive functions are more universal than achievement, we had hypothesized that academic achievement would be the primary moderator of parent/teacher agreement due to the higher academic demands at school. The fact that only reading skills, and not math skills, moderated agreement throws this into question. It is possible that reading skills face higher demands than math in school (i.e., in the school-age years, children are often “reading to learn”), but there is no convincing evidence that intellectual functioning faces increased demands at school compared to the home. Thus, the proposed mechanism of discrepancy, i.e. changes in actual child behavior based on situation-specific demands, was not fully upheld.

Our other proposed mechanism concerned altered parent/teacher perceptions. That is, cognitive abilities may be more salient to teachers compared to parents, leading to teachers rating children with lower cognitive abilities as struggling more overall. However, in both cases, the proposed mechanism assumed that discrepancies would be driven by lower cognitive abilities being associated with higher teacher, compared to parent, report of symptoms. Post-hoc analyses did not support this explanation. Instead, parents of children with lower estimated intellectual functioning (and, in some cases, reading skills) reported more externalizing and internalizing behaviors compared to teachers.

What, then, could be driving such patterns? Lower intellectual functioning and academic achievement are transdiagnostic risk factors for psychopathology in children (Kavish et al., 2020; Monzonís-Carda et al., 2024). Therefore, it may be that while children with lower intellectual ability demonstrate higher symptomatology in both environments, parents know their children better and are better suited to recognize such symptoms due to their greater time spent with their child, closer relationships, and lack of competing demands (i.e., managing a full classroom). Indeed, meta-analyses have indicated that parent-report tends to be more closely aligned with children’s self-report than teacher-report is, particularly for internalizing symptoms (Achenbach et al., 1987; De Los Reyes et al., 2015). Thus, higher parent report in the context of lower cognitive abilities may reflect reporter accuracy or the relationship between the informant and the child, rather than different behaviors across settings.

It is also possible that lower cognitive functioning still altered teachers’ perceptions, but in the opposite pattern than expected. That is, teachers may have been aware of children’s intellectual or academic struggles (e.g., through knowledge of the learning support services received by 22.62% of the sample) and therefore attributed behavior problems to learning difficulties, rather than psychopathology. This would align with the Attribution Bias Context (ABC) Model proposed by De Los Reyes and Kazdin (2005), in which attributions about the cause of a child’s behavior impact the way questionnaires are completed. However, it is unclear why a similar pattern did not occur with academic achievement in mathematics. Furthermore, the receipt of learning support services was assessed via parent-report, and it is therefore unknown whether teachers in the current study had knowledge of such services.

Both explanations of course make the assumption that the informant reporting the higher symptoms is the “correct” informant. Future research might consider obtaining objective measures of behavior across multiple settings to compare against parent/teacher report. This would aid in determining whether the directionality of effects is a result of objectively divergent behaviors or different perceptions or knowledge of the child. Trained third-party observer report, ideally in multiple settings (e.g., De Los Reyes et al., 2009) could capture more overt behaviors, and in older children, self-report ratings of internalizing symptoms may prove informative (Hope et al., 1999). Researchers have also proposed methods such as ecological momentary assessment (EMA) to obtain granular reports of behavior over time in multiple settings, which would be particularly useful for capturing differences in behavioral expression across environments and reducing perceptual biases such as recency or mood-congruent memory effects (Thunnissen et al., 2022).

Such research would provide a path forward for clinicians attempting to integrate or otherwise reconcile divergent reports and provide clinicians with guidelines on how or whether to weigh reports differently based on their knowledge of the child’s cognitive abilities. These findings also point to the importance of comprehensive assessment that is not only multi-informant, but multi-domain. That is, assessment should not only collect information on children’s functioning from multiple sources, including parents and teachers, but also assess children’s functioning in multiple domains, including cognitive abilities, in order to form a wholistic understanding of the child.

### Limitations and Future Directions

The present study has several notable strengths. Methodologically, the use of performance-based measures of cognitive abilities and a latent variable approach to modeling psychopathology allowed for the precise targeting of the constructs of interest, rather than merely capturing shared method variance. The inclusion of both internalizing and externalizing symptoms further broadened the scope of the findings, particularly given the lack of attention paid to internalizing symptom agreement in the literature. Nevertheless, the study’s design reflects a deliberate focus on non-demographic child characteristics, and, reflecting regional demographics, the study’s sample was relatively racially and ethnically homogenous. An important direction for future research would be to establish whether the observed patterns are stable across gender, race, ethnicity, and socioeconomic status. Given well-documented informant discrepancies (e.g., De Los Reyes & Kazdin, 2005; Lau et al., 2004) and systemic disparities in both mental health care and intellectual supports for racially-minoritized children (Marrast et al., 2016; Parish et al., 2013), it would be particularly important to examine the intersection of racial and ethnic identity with the current findings.

Furthermore, study recruitment oversampled for attention difficulties, and the sample contained a relatively high proportion of children diagnosed with ADHD (49.71%). Diagnoses of ADHD require symptoms to be present in multiple domains, which is often operationalized as elevated inattentive and hyperactive symptoms according to both parent and teacher report. Therefore, it is possible that the sample included a disproportionate number of children for whom parents and teachers at least somewhat agreed on externalizing behaviors. This may have decreased variability in parent/teacher agreement, therefore masking potential moderating effects on externalizing symptoms. The relationship between parent- and teacher-reported externalizing symptoms was still only moderate (*r* = .56), so this appears unlikely, but further replication in non-clinical samples, or samples weighted towards other presenting concerns, would strengthen the current findings.

Analytically, post-hoc analyses required categorizing participants into groups based on the direction of differences between parent and teacher report. Categorical analyses can force participants into artificial groups and exaggerate differences between individuals. Additionally, results may differ depending on the cut-off criteria used to form such groups. The current findings held even when the cut-off criteria was changed, which increases our confidence in these results, but the limitations of categorical analyses should be considered when interpreting findings.

Finally, the current study operationalized intellectual functioning using a two-subtest short-form of the WISC-IV. Although the two-subtest short-form demonstrates strong predictive validity of full-scale IQ (0.87), there can still be variation between estimated and full-scale IQ scores, and the use of short forms can magnify the effect of administration errors (Sattler & Dumont, 2004). Additionally, one of the two subtests used to calculate estimated IQ was vocabulary, which may share some conceptual overlap with the word reading subtest used to assess reading skills. WISC vocabulary assesses expressive word knowledge as a measure of verbal intelligence, whereas WIAT word reading assesses letter-sound knowledge and word identification, but both are related to overall literacy. In the current sample, estimated IQ was only moderately correlated with word reading (*r* = .59), and it is therefore unlikely that the two captured the same construct. However, future studies could consider full administration of assessments such as the WISC and WIAT for even more robust measures of intellectual and academic functioning to further explore the nuances of the current results.

## Conclusion

Multi-informant report is the gold standard for child clinical assessment; however, clinicians must often exercise clinical judgement when interpreting divergent reports from parents and teachers on internalizing and externalizing symptoms. The current study points to the role of cognitive abilities such as intellectual functioning and reading skills in parent/teacher disagreement. Our findings underscore the importance of comprehensive assessment that gathers information from multiple informants *and* in multiple domains. Critically, the value of such assessments lies not only in collecting information from diverse sources but also in meaningfully integrating such information. Research on the factors that contribute to informant discrepancies can provide clinicians and researchers with an empirically grounded basis for interpreting and synthesizing information to form a coherent, full-picture understanding of a child’s functioning.

## Data Availability

Data will be made available on request.

## Footnotes

1 Continuous difference scores were not used in analysis due to mathematical constraints discussed in the literature (Laird & De Los Reyes, 2013; Laird & Weems, 2011).
